# Association of Metabolic-Associated Fatty Liver Disease With Various Anthropometric Parameters in Pre-diabetes in Comparison With Diabetes and Control: A Single Tertiary Care Center Study

**DOI:** 10.7759/cureus.27130

**Published:** 2022-07-21

**Authors:** Karthik Kolluru, Anamika Giri, Sunil Kumar, Sourya Acharya, Sachin Agrawal, Anil Wanjari, Shilpa A Gaidhane

**Affiliations:** 1 Department of Medicine, Jawaharlal Nehru Medical College, Datta Meghe Institute of Medical Science (Deemed to be University), Wardha, IND; 2 School of Epidemiology and Public Health, Jawaharlal Nehru Medical College, Datta Meghe Institute of Medical Science (Deemed to be University), Wardha, IND

**Keywords:** body mass index, hba1c, pre-diabetes, diabetes, nafld

## Abstract

Introduction

Individuals with pre-diabetes and metabolic-associated fatty liver disease (MAFLD) have an increased risk of developing diabetes mellitus (type-2) when compared with individuals with pre-diabetes without MAFLD. Patients with any of the components of metabolic syndrome should be screened for the risk of MAFLD, as all its components are well correlated with the degree of liver fat content. In this research article, we have highlighted the association of MAFLD with various anthropometric parameters in pre-diabetes as compared to diabetes and normal individual.

Methods

In this cross-sectional study a total of 356 patients more than 18 years of age who meet the criteria for diabetes and pre-diabetes according to WHO, were enrolled. Anthropometric indices like body mass index (BMI), waist-hip ratio, waist to height ratio, and neck circumference were recorded. Patients underwent ultrasonography of liver and blood investigations like lipid profile, and liver function tests.

Results

The prevalence of MAFLD observed in this study was 44.1% in diabetics and 22% in pre-diabetics, compared to 9.2% in healthy controls. The ROC analyses showed that MAFLD predict pre-diabetes using the waist-hip ratio was higher in women compared to men (0.750 and 0.693 respectively). In men, the waist-hip ratio was followed by 0.648 for Neck Circumference, 0.646 for BMI, and 0.635 for waist-to-height ratio respectively, whereas the ROC analyses in women showed that other than waist-hip ratio, no other anthropometric index that had consistently higher AUC value.

Conclusion

Though there was an association between high BMI, waist-hip ratio, waist to height ratio, and neck circumference with MAFLD in pre-diabetes, it was not strongly associated as in the diabetic group.

## Introduction

Metabolic-associated fatty liver disease (MAFLD) has become a major public health problem, which is also the major causative factor of chronic liver disease across the globe [1-3]. MAFLD refers to the cluster of fatty changes in the liver in individuals with no alcohol consumption. The fatty changes can range from simple steatosis and may lead to extreme conditions, such as non-alcoholic steatohepatitis (NASH), cirrhosis, or hepatocellular carcinoma (HCC) [4-6].

Pathologically, MAFLD is classified into primary and secondary types, the primary causes being obesity, diabetes, dyslipidemia, insulin resistance, or metabolic syndrome. On the other hand, the secondary type of MAFLD is associated with endocrine diseases, Total parenteral nutrition, intake of some drugs ( amiodarone, perhexiline,4,4’-diethylaminoethoxyhexestrol), pancreatoduodenal resection, etc. [3-5].

The predisposing factors of MAFLD are metabolic syndrome and its associated conditions are type 2 diabetes, obesity, and dyslipidemia. The association between type 2 diabetes mellitus and MAFLD has been recognized recently. It was observed that the prevalence of MAFLD was higher in type 2 DM patients compared to their non-diabetic counterparts [7,8]. The critical factor that induces increased lipolysis in peripheral adipose tissue and increases the absorption of fatty acids by hepatocytes is supposed to be insulin resistance. Hyperinsulinemia as a result of insulin resistance also increases the fatty acid content of hepatocytes. The resulting condition is an increase in fatty acids and triglycerides in the hepatocytes which causes steatosis [9,10].

According to the SPRINT study, a multicenter study conducted in 101 cities in India, the prevalence of MAFLD among 25-84-year-old T2DM patients was 56.5%. The prevalence was highest in northern states (72.4%), while the lowest prevalence was recorded in the western part of India [3,4,9].

Assessment of liver histology by liver biopsy is considered the gold standard to diagnose fatty liver. In spite of this fact, it is not suitable for population-based screening due to the risks, cost of the test, and uncertain advantages to asymptomatic patients. In such situations, the elevation of liver enzymes and supported liver sonography are helpful during a routine examination.

Only a few studies had been reported in India [4,9]. This may be because the condition was recognized only in recent years, MAFLD is assumed to be a benign and non-progressive condition and high prevalence of viral hepatitis in India masked the priority of MAFLD. In South Asian countries, especially India, the prevalence of diabetes is very high with a high frequency of insulin resistance. Individuals with pre-diabetes and MAFLD have an increased risk of developing diabetes mellitus (type-2) when compared with individuals with Pre-diabetes without MAFLD.

## Materials and methods

This cross-sectional study was accomplished under the Medicine department from October 2018 to July 2020 at a rural teaching hospital in central India after clearance from the institutional ethical committee with an approval letter numbered DMIMS (DU)/IEC/2018-19/7545.

 All patients coming to OPD/IPD in the age group of 18 and above diagnosed and fulfilling WHO criteria for pre-diabetes and diabetes are included in the study. According to WHO diabetics were defined as people with fasting plasma glucose values of ≥ 126 mg/dL, postprandial values of more than 200 mg/dL, or HbA1c ≥ 6.5%; or random blood glucose of 200 mg/dL in the presence of signs and symptoms.

Pre-diabetics were defined as cases with fasting serum glucose levels between 110 and 125 mg/dL and/or two-hour plasma glucose levels after 75gmOGTT between 140 mg/dL and 199 mg/dL according to WHO criteria.

Patients who consume alcohol, have other liver disorders, such as hepatitis, liver abscess, malignancies, and patients having derangement of hepatic function due to some other febrile illnesses/disease are excluded.

The study population included 100 pre-diabetic and diabetic patients, and 100 age and sex-matched healthy subjects who were the controls. All the study subjects underwent thorough physical general and systemic examination. For each study participant, anthropometric measurements were reported for height, weight, waist circumference, hip circumference, Waist-hip ratio (WHR), waist to height ratio, and neck circumference. BMI was known to be an obesity indicator and was measured by dividing weight in kilograms by the height square in meters. With the study subject in standing place at the level of the umbilicus, between the lowest rib and the iliac crest, and at the level of the great trochanter respectively, the waist and Hip circumference were calculated. The WHR was calculated as an indication of fat accumulation. The WHR was measured with the ratio of WC to HC calculated. WHR >0.95 in males and >0.85 in females is considered obesity according to WHO. Waist to height ratio of >0.53 in males and >0.49 in females is considered obesity. Neck circumference (cm) was measured in the middle of the neck, between the middle anterior neck and middle cervical spine, within 1 mm, using a calibrated plastic tape. NC > 37 cm in men and >34 cm in women was considered obesity. All patients will undergo an ultrasound (USG) of the abdomen to detect fatty changes in the liver, performed by an experienced radiologist and then confirmed by another senior radiologist. It will be performed using a high-resolution B-mode ultrasonography system pro sound Alpha 7 with curved linear arrays mid-frequency probe of 3-5 MHz on ARIETTA 65 machine.

Diagnosis of MAFLD is made by the presence of an ultrasonographic pattern consistent with increased echogenicity, i.e., evident ultrasonographic contrast between hepatic and renal parenchyma.

Standard ultrasonographic grading of MAFLD is classified into three grades. Grade 1 has mild increase in fine echoes in hepatic parenchyma with normal appearance diaphragm and intrahepatic vessel borders. Grade 2 has moderate increase in fine echoes with a slight abnormal appearance of the intrahepatic vessels and diaphragm. Grade 3 has A marked increase in fine echoes with the disappearance of intrahepatic vessel borders, diaphragm, and posterior part of the right hepatic lobe.

**Figure 1:**
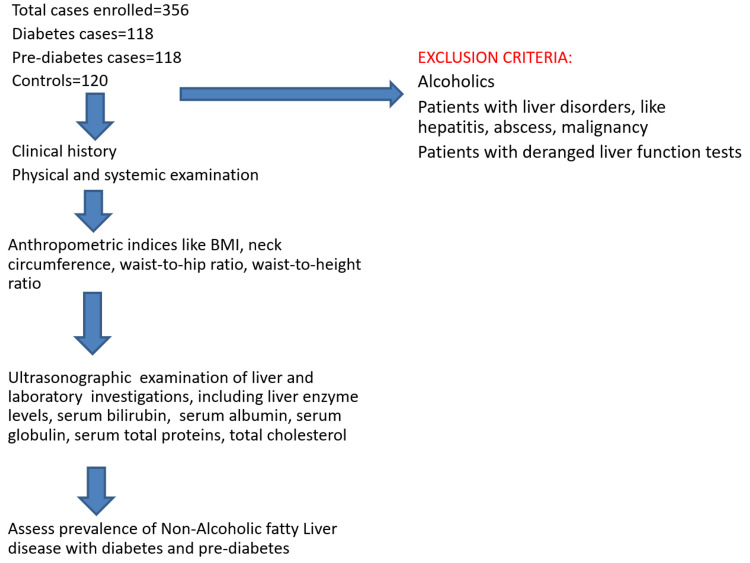
Study Process

Using the ultrasonographic findings, patients were categorized as with MAFLD and without MAFLD. The flow chart of the study is shown in Figure 1.

Statistical analysis

Statistical analysis was done by using descriptive and inferential statistics using the Chi-square test and Student’s unpaired t-test and software used in the analysis were SPSS 22.0 version (IBM Corp., Armonk, NY) and Graph Pad Prism 6.0 version and p<0.05 is considered as the level of significance.

## Results

Out of 356 cases, the majority of study subjects (56.8%) in the diabetes group were >60years, while most of the patients in the pre-diabetes (43.2%) and control (44.2%) were in the age range of 40-60 years. This difference between the various groups in terms of distribution of age was statistically significant (p = 0.008). Elevated serum triglycerides were highest in the diabetic group (37.3%) followed by pre-diabetics (21.2%). Other baseline characteristics are shown in Table 1.

**Table 1:** Baseline characteristics ***Significant at p<0.05, 1: Kruskal Wallis Test, 2: Chi-Squared Test, 3: Fisher's Exact Test

Parameters		Group						P-value	
		Diabetes (n = 118)		Pre-Diabetes (n = 118)		Control (n = 120)			
Age (Years)***		54.58 ± 14.53		51.36 ± 13.93		52.38 ± 14.12		0.025^1^	
Age***								0.008^2^	
20-40 Years		16 (13.6%)		18 (15.3%)		26 (21.7%)			
40-60 Years		35 (29.7%)		51 (43.2%)		53 (44.2%)			
>60 Years		67 (56.8%)		49 (41.5%)		41 (34.2%)			
Gender***								0.026^2^	
Male		70 (59.3%)		89 (75.4%)		84 (70.0%)			
Female		48 (40.7%)		29 (24.6%)		36 (30.0%)			
Body mass index (Kg/m2)***		24.08 ± 2.80		22.62 ± 2.77		20.96 ± 2.11		<0.001^1^	
Waist-Hip Ratio									
Male	0.95±0.032		0.90±0.05		0.89±0.05				
Female	0.95±0.034		0.95±0.05		0.89±0.05				
Waist-Height Ratio								<0.001^2^	
Male		53.15±4.1		58.33±4.9		54.97±5.4			
Female		53.15±4.1		58.25±4.9		54.45±5.1			
Neck Circumference								0.001^2^	
Male		32.13±1.85		32.93±1.09		32.12±1.47			
Female		32.13±1.85		32.93±1.10		32.02±1.45			
Serum high density lipoprotein(mg/dL)***								<0.001^2^	
Male		52.3±19.7		46.2±14.2		52.04±8.4			
Female		52.5±19.5		46.2±14.1		51.20±7.6			
Serum Triglycerides		150.67 ± 29.9		147.77 ± 42.58		133.74 ± 16.47		<0.001^1^	
Metabolic associated fatty liver disease Grade (USG)***								<0.001^3^	
Absent		66 (55.9%)		92 (78.0%)		112 (93.3%)			
Grade 1		34 (28.8%)		19 (16.1%)		8 (6.7%)			
Grade 2		15 (12.7%)		7 (5.9%)		0 (0.0%)			
Grade 3		3 (2.5%)		0 (0.0%)		0 (0.0%)			
Metabolic associated fatty liver disease (Present)***		52 (44.1%)		26 (22.0%)		11 (9.2%)		<0.001^2^	

Cases of MAFLD are highest among diabetic group (44.1%) followed by pre-diabetic group (22.0%) as shown in Table 2.

**Table 2:** Distribution of patients with MAFLD according to group (n = 356)

Metabolic associated Fatty Liver Disease	Group				Chi-Squared Test	
	Diabetes	Pre-Diabetes	Control	Total	χ2	P Value
Present	52 (44.1%)	26 (22.0%)	11 (9.2%)	89 (25.0%)	39.479	<0.001
Absent	66 (55.9%)	92 (78.0%)	109 (90.8%)	267 (75.0%)		
Total	118 (100.0%)	118 (100.0%)	120 (100.0%)	356 (100.0%)		

Overall prevalence of MAFLD among study subjects was 25%, out of which, 44.1% were diabetics followed by 22% from pre-diabetes group while only 9.2% controls had MAFLD.

**Figure 2:**
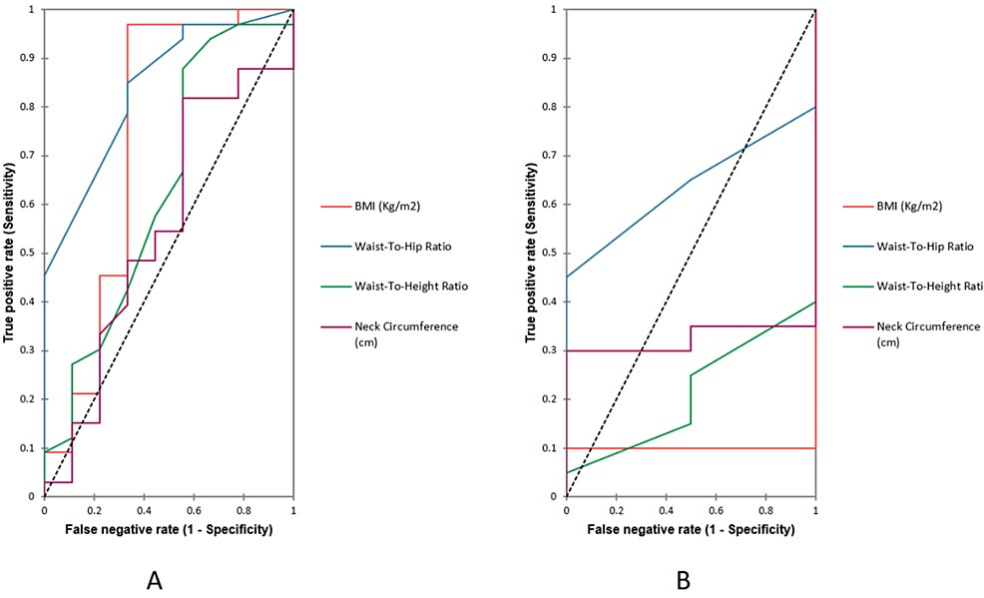
ROC of patients with MAFLD in pre-diabetes. (A) Males. (B) Females

The AUC of the ROC analyses showed that the area under the curve to predict pre-diabetes using WHR was higher in females compared to males (0.750 and 0.693, respectively). In males, WHR was followed by 0.648 for neck circumference, 0.646 for BMI, 0.635 for waist-to-height ratio, whereas the AUC of the ROC analyses in females showed that other than WHR, no other anthropometric index that had consistently higher AUC value. WHR in males had highest AUC as shown in Figure 2.

**Figure 3:**
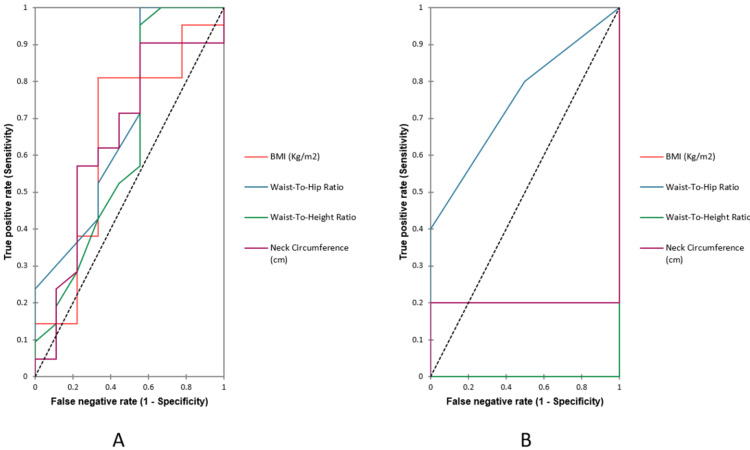
ROC of patients with MAFLD in diabetes. (A) Males. (B) Females.

The AUC of the ROC analyses showed that the area under the curve to predict diabetes was higher in males compared to females. Highest AUC was observed in males for WHR (0.840 in males and 0.638 in females) followed by BMI (0.737 in males and 0.1 in females), waist-height ratio (0.631 in males and 0.213 in females) and neck circumference (0.552 in males and 0.325 in females) as shown in Figure 3.

**Figure 4:**
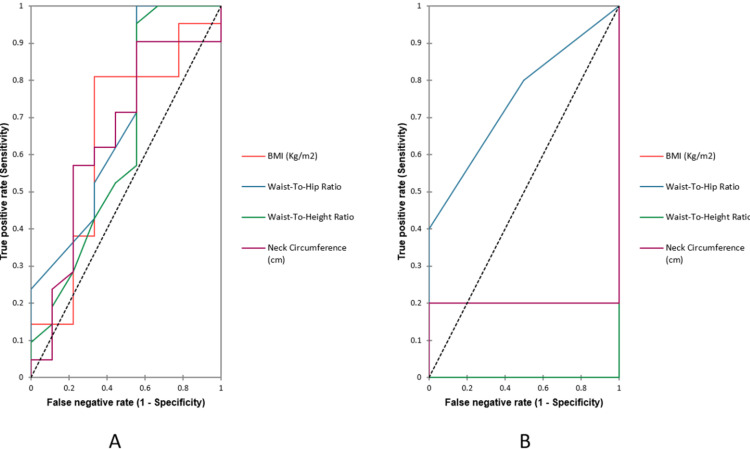
ROC of patients with MAFLD in control. (A) Males. (B) Females.

The AUC of the ROC analyses showed that the area under the curve to predict controls was higher for WHR in females compared to males (0.750 and 0.500, respectively). In males, WHR was followed by 0.6571 for BMI, 0.536 for neck circumference and 0.512 for waist-to-height ratio, whereas the AUC of the ROC analyses in females showed that other than WHR, no other anthropometric index that had consistently higher AUC value as shown in Figure 4.

## Discussion

The prevalence of MAFLD observed in this study was 44.1% in diabetics and 22% in pre-diabetics, compared to 9.2% in healthy controls, based on ultrasound findings. Various anthropometric indices like BMI, WHR; waist-height ratio, and neck circumference were calculated in pre-diabetes subjects in comparison with diabetics and control. MAFLD in Pre-diabetes is rarely explored, so only limited literature is available for our current study [11-16].

In the present study, the AUC of the ROC analyses showed that the area under the curve to predict pre-diabetes using WHR was higher in females compared to males (0.750 and 0.693, respectively) [17]. Few studies in India had also shown a similar correlation between MAFLD and diabetes as well as prediabetes [18-21]. A concurrent study which was conducted by the University of Oradea in Romania showed 93.20±7.24 with a P-value of 0.02.7. In a cross-sectional study of the Ethiopian population by considering anthropometric indices in pre-diabetes, the AUC of the ROC analysis showed 0.57 and 0.54 in females and males, respectively [8]. In our study among the pre-diabetic group, WHR in females had the highest AUC, and WHR in males had the highest sensitivity.

In males, the WHR was followed by 0.648 for neck circumference whereas in females; neck circumference was not higher than WHR. Till now, no literature was found on neck circumference in the MAFLD population with pre-diabetes.

BMI in males showed higher AUC in all the three groups (0.64, 0.3, 0.57 in pre-diabetes, diabetes, and controls), while in females, AUC for BMI was negligible (0, 0.1, 0 in Pre-diabetes, Diabetes, and controls). A study conducted in Romania among Pre-diabetics with MAFLD showed 26.04±3.56 with a P-value of 0.09.7 Another cross-sectional study had shown the pre-diabetes population as 0.57 and 0.59 in males and females with MAFLD association [22,23].

In the male, the waist-height ratio was 0.635, whereas the AUC of the ROC analyses in females showed that other than the WHR, no other anthropometric indices were consistently higher AUC value. Woldegebriel et al. conducted a study on the Ethiopian population in pre-diabetes with the association of anthropometric parameters, AUC depicts 0.61 in males and 0.58 in females [8].

WHR was significantly higher in diabetic male subjects (0.840 in males and 0.638 in females) when compared with Pre-diabetic and control male subjects, where the majority had normal ratios (males - 0.693 and 0.5000, females - 0.750 and 0.750). The finding of this study was not concurrent with the Ethiopian population-based - cross-sectional study which showed 0.57 in males and 0.67 in female subjects in diabetics when compared with Pre-diabetics (0.54 and 0.57 in males and females respectively) [8].

Limitations

Liver biopsy is known to be the gold standard investigation for MAFLD diagnosis and staging. However, in broad epidemiological studies, liver biopsy is not certainly applied; USG abdomen is considered to be the most sensitive investigation in clinical practice for diagnosing NAFLD.

## Conclusions

Anthropometric indices, like BMI, WHR, waist-height ratio, and neck circumference, were higher than normal in patients with MAFLD. Diabetics show a significant rise in different anthropometric parameters, whereas pre-diabetics show a relative increase when compared with control. Our study concludes with pre-diabetes as an emerging burden of health issue globally, for which anthropometric parameters correlate with the clinical diagnosis but are not strongly associated as in the diabetic group.
